# Optimizing biodiesel production in marine *Chlamydomonas* sp. JSC4 through metabolic profiling and an innovative salinity-gradient strategy

**DOI:** 10.1186/1754-6834-7-97

**Published:** 2014-06-24

**Authors:** Shih-Hsin Ho, Akihito Nakanishi, Xiaoting Ye, Jo-Shu Chang, Kiyotaka Hara, Tomohisa Hasunuma, Akihiko Kondo

**Affiliations:** 1 Organization of Advanced Science and Technology, Kobe University, 1-1 Rokkodai, Nada-ku, Kobe 657-8501, Japan; 2 Department of Chemical Science and Engineering, Graduate School of Engineering, Kobe University, 1-1 Rokkodai, Nada-ku, Kobe 657-8501, Japan; 3 Department of Chemical Engineering, National Cheng Kung University, No.1 University Road, Tainan 701, Taiwan; 4 Research Center for Energy Technology and Strategy, National Cheng Kung University, No.1 University Road, Tainan 701, Taiwan; 5 Center for Bioscience and Biotechnology, National Cheng Kung University, No.1 University Road, Tainan 701, Taiwan; 6 Biomass Engineering Program, RIKEN, 1-7-22 Suehiro-cho, Tsurumi-ku, Yokohama, Kanagawa 230-0045, Japan; 7 Department of Food Bioscience and Technology, College of Life Sciences and Biotechnology, Korea University, Seongbuk-gu, Seoul 136-713, Republic of Korea

**Keywords:** Microalgae, *Chlamydomonas* sp, Lipids, Biodiesel, Metabolite profiling, Salinity-gradient operation, Nitrogen depletion

## Abstract

**Background:**

Biodiesel production from marine microalgae has received much attention as microalgae can be cultivated on non-arable land without the use of potable water, and with the additional benefits of mitigating CO_2_ emissions and yielding biomass. However, there is still a lack of effective operational strategies to promote lipid accumulation in marine microalgae, which are suitable for making biodiesel since they are mainly composed of saturated and monounsaturated fatty acids. Moreover, the regulatory mechanisms involved in lipid biosynthesis in microalgae under environmental stress are not well understood.

**Results:**

In this work, the combined effects of salinity and nitrogen depletion stresses on lipid accumulation of a newly isolated marine microalga, *Chlamydomonas* sp. JSC4, were explored. Metabolic intermediates were profiled over time to observe transient changes during the lipid accumulation triggered by the combination of the two stresses. An innovative cultivation strategy (denoted salinity-gradient operation) was also employed to markedly improve the lipid accumulation and lipid quality of the microalga, which attained an optimal lipid productivity of 223.2 mg L^-1^ d^-1^ and a lipid content of 59.4% per dry cell weight. This performance is significantly higher than reported in most related studies.

**Conclusions:**

This work demonstrated the synergistic integration of biological and engineering technologies to develop a simple and effective strategy for the enhancement of oil production in marine microalgae.

## Background

Fossil fuels now contribute most of the global energy demand, but these fuels are directly associated with the greenhouse effect and environmental pollution [1]. Oil reserves may run out after 2050 due to the fast growth in fossil fuel-requiring human activities worldwide [2,3]. Therefore, huge efforts are being made in developing CO_2_ fixation and reduction technologies and in finding alternative and renewable energy sources [4]. Among those attempts, biodiesel has received significant attention since it is made from nontoxic and biodegradable materials, and its use leads to a huge decrease in the emissions of greenhouse gases (such as CO_2_) and air pollutants [5]. However, to produce enough biodiesel from oleaginous crops (such as soybean, palm, and rapeseed) to supply the existing demand for transportation in the United States alone, displacement of around 50% of its total cropland would be necessary. This would cause severe problems such as food shortages [6]. Therefore, there is an urgent need to identify more effective and sustainable oil feedstocks for making biodiesel to meet the global demand.

Microalgae are considered a renewable alternative biodiesel feedstock due to their high growth rate, relatively high lipid content, and excellent CO_2_ fixation ability compared to other candidates [5,7]. Under appropriate environmental stress some microalgal species such as *Chlorella emersonii*, *Chlorella minutissima*, *Chlorella protothecoides*, *Chlorella vulgaris*, *Neochloris oleabundans*, and *Dunaliella tertiolecta* can accumulate up to 50 to 70% of lipid per dry weight of biomass, and the resulting lipid composition is suitable for making biodiesel [7-9]. However, for large-scale microalgal cultivation, a tremendous amount of water is required. This would raise the problem of a shortage of available water resources if freshwater is used to grow microalgae without efficient recycling of the spent water [10,11]. Therefore, marine microalgal strains that could grow in brackish water or seawater have clear advantages over freshwater algae in terms of water usage and could be a more reliable and economic feedstock for microalgal-based biodiesel production. In addition, some critical biological features such as growth rate, lipid content, and lipid composition are crucial for justifying the feasibility of using microalgae to produce lipids for biodiesel synthesis. Thus, selecting a marine candidate with rapid growth, high lipid content, and appropriate lipid composition is essential. Several marine microalgae have been considered as potential biodiesel producers due to their characteristics of high salt tolerance and high lipid content. These species include *Chlorella sorokiniana*[12], *Nannochloropsis* sp. F&M-M24 [13], *Nannochloropsis gaditana*[10], *D. tertiolecta* ATCC30929 [14], and *C. protothecoides*[15].

The production of microalgal lipids is often triggered by environmental stress such as nitrogen depletion [7]. However, operation under stress conditions is always associated with relatively low cell growth, thereby leading to low lipid productivity [5,16,17]. Many studies have reported that growth rate and lipid accumulation in microalgae are significantly influenced by culture conditions (such as irradiance, salt concentration, and medium selection) [14,18,19] and operational strategies (such as two-stage cultivation, semi-batch cultivation, or continuous cultivation) [5,10,12,20]. Although the information provided in the literature indicates that lipid production from microalgae could be improved by applying suitable operational strategies and conditions, a systematic analysis on how to optimize lipid productivity and on the metabolic mechanism behind the improvement in lipid accumulation is still lacking. In particular, lipid production using marine microalgae has been little studied.

Metabolic profiling analysis has proven to be a powerful tool for gaining insights into functional biology in recent years [21]. The comprehensive analysis of a wide range of metabolites in microorganisms using high-sensitivity mass spectrometry techniques makes it possible to identify targeted metabolites that play important roles in specific biological processes [22]. This makes metabolomics a perfect approach to explore why lipids are accumulated under particular stress conditions. However, metabolomics tools have not yet been widely applied to investigating lipid-producing microalgae. Therefore, we used applied metabolomics to observe the main metabolite profiles of the target lipid-producing microalgae during cultivation under the combined environmental stresses of salinity and nitrogen depletion.

In this study, a newly isolated microalgal strain identified as *Chlamydomonas* sp. JSC4 was used as a candidate oil producer. A variety of culturing conditions, including various media, salinity, and nitrogen depletion were examined to optimize lipid productivity. In addition, the metabolic profile of *Chlamydomonas* sp. JSC4 under salt stress and nitrogen depletion was monitored to identify the intercorrelations among salinity, nitrogen depletion, and lipid synthesis. Finally, various engineering strategies (namely two-stage and salinity-gradient operations) were applied to optimize the lipid productivity of the JSC4 strain. In particular, salinity-gradient operation is a novel method that is, in principle, applicable to any other marine microalgal strain as long as it shows some salinity tolerance. Thus, this study demonstrates an effective combination of biological and engineering technologies enabling the rapid accumulation of lipid in a target marine microalga to meet the demand for biodiesel feedstock.

## Results and discussion

### Effect of medium composition on cell growth, CO_2_ fixation, and lipid accumulation of *Chlamydomonas* sp. JSC4

**Figure 1:**
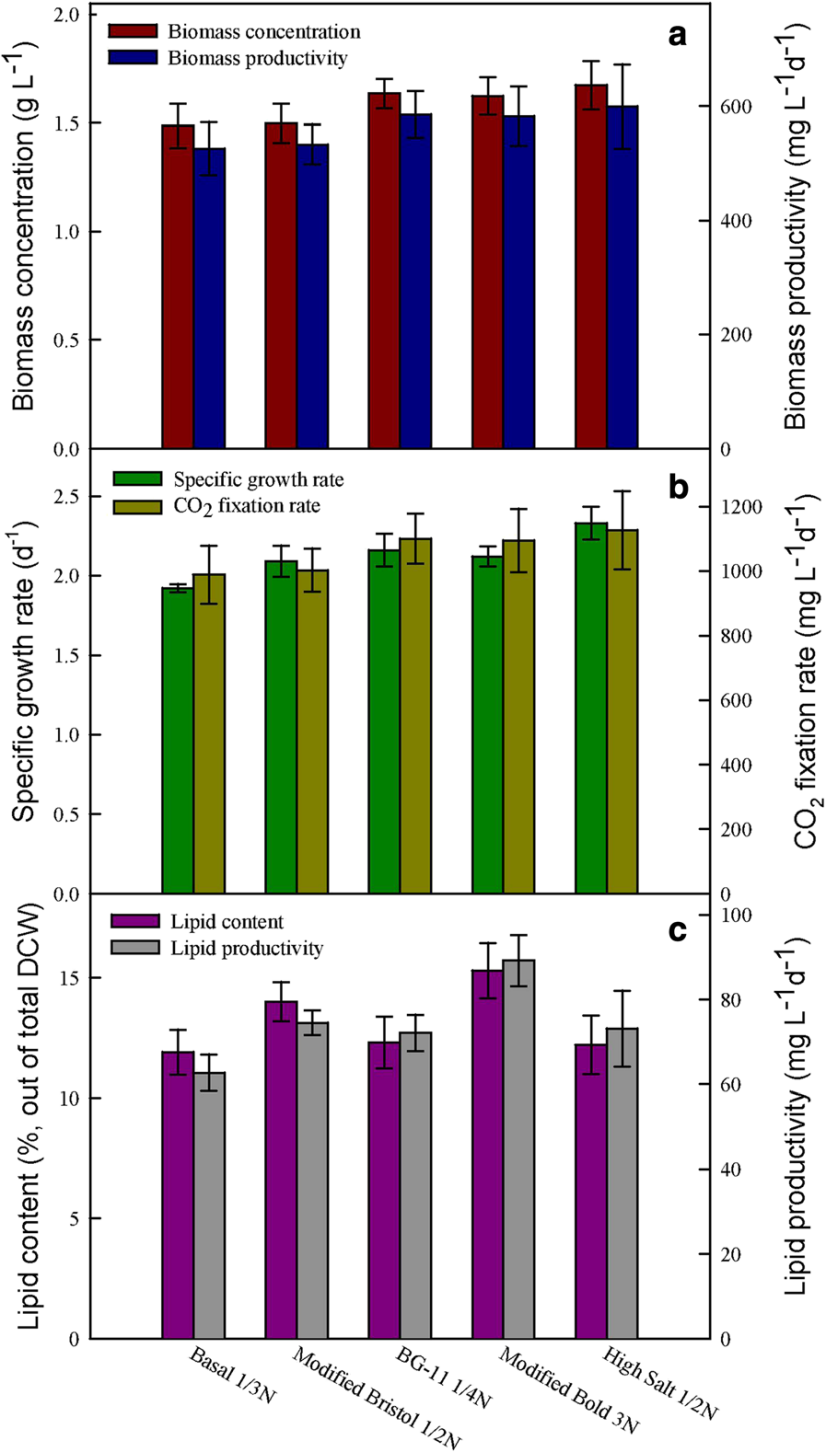
**Performance of *Chlamydomonas *sp. JSC4 cultivated on different growth media based on cell growth, CO**_**2 **_**fixation, and lipid accumulation. (a)** Biomass concentration (g L^-1^) and biomass productivity (mg L^-1^ d^-1^), **(b)** specific growth rate (d^-1^) and CO_2_ fixation rate (mg L^-1^ d^-1^), and **(c)** lipid content (%) and lipid productivity (mg L^-1^d^-1^). Error bars indicate standard deviation of three replicated experiments. (Light intensity = 200 μmol m^-2^ s^-1^; CO_2_ aeration = 2.0%; CO_2_ flow rate = 0.05 vvm).

The growth rate and cellular composition of microalgae varies significantly depending on the composition of their growth medium [18]. To select a suitable medium for the growth and lipid accumulation of *Chlamydomonas* sp. JSC4 after pre-culturing on Modified Bold 3 N medium, five commonly used media (basal medium 1/3 N, Modified Bristol 1/2 N medium, BG11 1/4 N medium, Modified Bold 3N medium, and high salt 1/2 N medium) were used to grow the JSC4 strain for lipid accumulation. The nitrogen and sea salt concentrations in each medium were fixed at 4.4 mM and 2%, respectively, to normalize the effects of the medium composition on cell growth and lipid accumulation. All cultures were grown for around two days under nitrogen-rich conditions. As shown in Figure 1, the biomass concentration, specific growth rate, and CO_2_ fixation rate of the JSC4 strain were quite similar under the five media examined, suggesting that the strain can grow well within a wide range of major nutrients and trace metals, showing a strong possibility of growing this strain in an outdoor system using wide range of water resources.

Figure 1 also indicates that different media slightly influenced the lipid content of the microalgal strain, as the highest lipid content (15.3%) and lipid productivity (89.2 mg L^-1^d^-1^) were obtained when it was grown on Modified Bold 3 N medium. According to these results, Modified Bold 3 N medium was used in the following experiments. It is commonly believed that lipid productivity is the most important indicator for microalgal lipid production, since higher lipid content would benefit downstream extraction. The total cost of chemicals in the medium is also a vital consideration for the practical scale-up of cultivation. As indicated in Table 1, mediums Modified Bold 3 N and BG11 1/4 N cost the least among the five media examined and are thus the most economic medium for microalgal cultivation. However, under the cultivation conditions used in these tests, the highest lipid content and lipid productivity (15.3% and 89.2 mg L^-1^d^-1^, respectively) were unsatisfactory compared to other relevant studies [12,13,16]. Further improvement in the lipid production of this strain was necessary and is described in the following sections.

**Table 1:** **Effects of medium composition on cell growth and lipid production of *Chlamydomonas *sp. JSC4**

** Medium**	**Biomass productivity (mg L**^**-1**^**d**^**-1**^**)**	**Lipid content (%)**	**Lipid productivity (mg L**^**-1 **^**d**^**-1**^**)**	**CO**_**2 **_**fixation rate (mg L**^**-1 **^**d**^**-1**^**)**	Nutrient price* ($ per ton)
Basal 1/3 N	375 ± 22	24.1 ± 1.9	90 ± 8	705 ± 41	40.17
Modified Bristol 1/2 N	352 ± 16	35.7 ± 1.4	126 ± 7	661 ± 29	6.44
BG-11 1/4 N	412 ± 19	36.0 ± 1.7	148 ± 7	774 ± 36	5.25
Modified Bold 3 N	378 ± 23	41.1 ± 2.2	155 ± 8	710 ± 43	5.25
High salt 1/2 N	356 ± 14	34.8 ± 1.7	124 ± 6	670 ± 26	19.74

The values are the mean ± standard deviation of three replicated experiments. (Light intensity = 200 μmol m^-2^ s^-1^; CO_2_ aeration = 2.0%; CO_2_ flow rate = 0.05 vvm).

*The price of media was evaluated using industrial-gradient nutrients purchased from local manufacturers.

### Effect of nitrogen depletion on lipid accumulation of *Chlamydomonas* sp. JSC4

**Figure 2:**
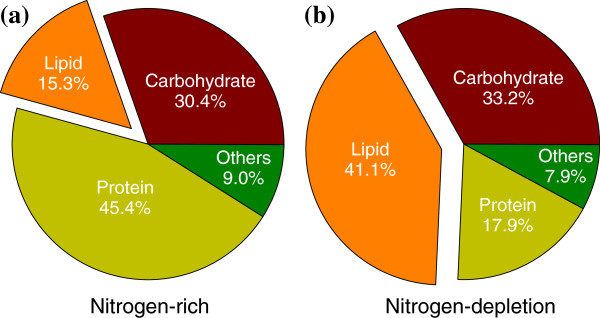
**Biochemical composition of *Chlamydomonas *sp. JSC4 cultivated on Modified Bold 3 N medium under (a) nitrogen-rich (70 to 80% nitrogen consumed) and (b) nitrogen-depleted conditions (three-day nitrate depletion).** (Light intensity = 200 μmol m^-2^ s^-1^; CO_2_ aeration = 2.0%; CO_2_ flow rate = 0.05 vvm).

Although the lipid content of a microalga appears to be species-specific, most oleaginous microalgae produce only a small quantity of lipids under conditions favorable for their growth [3]. To enhance lipid accumulation in microalgae, environmental stress (in particular, nitrogen depletion) is often employed to stimulate lipid synthesis [23]. Hu *et al.* reported that of all the nutrients evaluated the nitrogen source is the most critical factor affecting lipid synthesis in microalgae [23]. A number of studies have demonstrated that nitrogen depletion leads to a significant increase in lipid content in microalgae, which is accompanied with a drop in protein content. It is likely that the stress of nitrogen depletion forces the microalgae to transform protein or peptides to energy-rich compounds (such as lipids or carbohydrates) [2,24]. Thus nitrogen depletion was also used to promote lipid production in the JSC4 strain. As shown in Figure 2, when the microalga was grown on Modified Bold 3 N medium, a three-day nitrogen depletion resulted in a decrease in the protein content from 45.4 to 17.9%, whereas the lipid content increased dramatically from 15.3 to 41.1%. However, when using the other four media to grow strain JSC4, a three-day nitrogen depletion resulted in an improvement in the lipid content, which increased to 24.1, 35.7, 36.0, and 34.8% for basal 1/3 N, Modified Bristol 1/2 N, BG11 1/4 N, and high salt 1/2 N medium, respectively (Table 1). These results indicate that nitrogen depletion can dramatically increase the lipid content of strain JSC4 but has less impact on carbohydrate accumulation (data not shown). When microalgae undergo environmental stress like nitrogen depletion, either the lipid or carbohydrate metabolic pathway is often triggered, but to which energy-rich products (lipids or carbohydrates) the carbon is predominantly allocated is species dependent [24]. There also appears to be competition between lipid and starch synthesis when microalgae undergo nitrogen depletion [24].

### Effect of nitrogen depletion on the quality of lipids from *Chlamydomonas* sp. JSC4 for biodiesel

The quality of biodiesel can be based on its ratio of saturated to unsaturated fatty acids (FAs) [25]. The saturated FA content in biodiesel can increase its resistance to oxidation under high ambient temperatures, while unsaturated FAs can maintain fluidity at low temperatures [26]. A similar amount of saturated and unsaturated fatty acids is important in order to obtain biodiesel with appropriate properties at low temperature and high oxidative stability [16]. The FA profile is affected by environmental stress arising from nutrient limitations, temperature, salinity, and light intensity [15,23]. Among these stresses nitrogen depletion is the most critical factor affecting the lipid metabolism of microalgae and its effects have been observed in numerous microalgal species [7,9,23]. As indicated in Table 2, when undergoing nitrogen depletion the lipid accumulated in strain JSC4 tended to contain more oleic acid (C18:1), while the linolenic acid (C18:3) content sharply decreased. In terms of biodiesel properties, oils containing a higher percentage of oleic acid provide better oxidation stability with a suitable cold filter plugging point in cold weather [27]. In addition, based on the European Biodiesel Standard EN14214, the content of linolenic acid (C18:3) is limited to a maximum of 12% [28]. Thus, the lipids produced by *Chlamydomonas* sp. JSC4 seem to have suitable qualities for making biodiesel.

**Table 2:** **Fatty acid composition of *Chlamydomonas *sp. JSC4 cultured under nitrogen-rich and nitrogen-depleted conditions, with comparison to soybean oil (SBO)-based biodiesel**

	**Fatty acid composition (%)**		
	**2.0% sea salt**		**SBO-based biodiesel **[29]
	**Nitrogen rich**	**Nitrogen depletion**	
Palmitic acid (C16:0)	25.8 ± 0.6	27.6 ± 0.3	10.7
Palmitoleic acid (C16:1)	1.1 ± 0.0	3.2 ± 0.1	0.0
Stearic acid (C18:0)	4.0 ± 1.5	3.1 ± 0.1	4.4
Oleic acid (C18:1)	9.1 ± 0.0	26.6 ± 0.6	23.3
Linoleic acid (C18:2)	21.7 ± 0.0	25.3 ± 0.3	54.1
Linolenic acid (C18:3)	14.4 ± 0.4	5.4 ± 0.1	7.5
Saturated fatty acid	29.8 ± 1.3	30.7 ± 0.5	15.1
Monounsaturated fatty acid	10.2 ± 0.4	29.7 ± 0.5	23.3
Polyunsaturated fatty acid	36.1 ± 0.9	30.7 ± 0.4	61.6
C16 & C18 groups	76.1 ± 0.9	91.1 ± 1.3	100.0

The values are the mean ± standard deviation of three replicated experiments. (Light intensity = 200 μmol m^-2^ s^-1^; CO_2_ aeration = 2.0%; CO_2_ flow rate = 0.05 vvm).

Moreover, in contrast to soybean oil, strain JSC4 showed a significantly higher saturated FA content and a lower polyunsaturated FA content, as shown in Table 2. In general, a higher extent of saturation in lipids results in greater viscosity and density of the derived biodiesel, while lower percentages of polyunsaturated FAs can increase the oxidation stability and also result in an appreciable cold filter plugging point in cold regions [30]. Thus, due to having suitable FA profiles in its lipids, *Chlamydomonas* sp. JSC4 seems to be a promising feedstock for biodiesel production.

### Combined effects of salinity and nitrogen limitation on lipid accumulation and CO_2_ fixation of *Chlamydomonas* sp. JSC4

Many microalgae species can synthesize a large amount of lipids as storage products in response to unfavorable environmental conditions for growth, such as high light intensity [31], nutrient (mainly nitrogen) depletion [5], and high salinity [14]. In particular, for marine microalgae, salinity is an important factor affecting lipid content. Increases in salinity may result in a significant increase in the lipid content of several microalgal species, including *Chlorococcum* sp. [27], *Dunaliella* sp. [14], *Botryococcus braunii*[32], *Chlorella protothecoides*[15], and *Nannochloropsis* sp. [31]. A salinity control strategy to promote lipid production of microalgae is very easy to operate but has been little studied for effects on lipid accumulation when combined with nitrogen limitation. Since strain JSC4 is a marine microalga, it is of great interest to know whether salinity control could further enhance its lipid productivity, and more importantly to understand the possible synergistic effects when both salinity control and nitrogen depletion are employed. In addition, it is also necessary to explore appropriate timing in harvesting the cultured microalgae so that optimal lipid production efficiency can be obtained.

**Figure 3:**
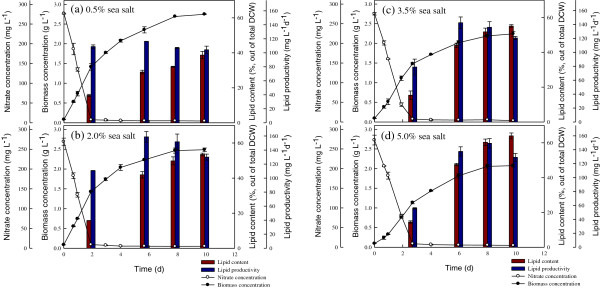
**Time course profiles of nitrate concentration, lipid content, and lipid productivity during growth of *Chlamydomonas *sp. JSC4 on Modified Bold 3 N medium under the sea salt concentration of (a) 0.5%, (b) 2.0%, (c) 3.5%, and (d) 5.0%.** Error bars indicate standard deviation of three replicated experiments. (Light intensity = 200 μmol m^-2^ s^-1^; CO_2_ aeration = 2.0%; CO_2_ flow rate = 0.05 vvm). DCW, dry cell weight.

In this study, the JSC4 strain cells were cultivated under various sea salt concentrations (0.5, 2.0, 3.5, and 5.0%) for 10 days under nitrogen-depleted conditions with regular monitoring of biomass production, nitrate concentration, lipid content, and lipid productivity. As shown in Figure 3 and Table 3, the final biomass concentration decreased slightly when raising the sea salt concentration from 0.5 to 5.0%, suggesting that high salt stress only slightly inhibits cell growth, due to the salt tolerance of strain JSC4. Inhibition of cell growth of microalgae by high salinity is commonly observed. For instance, Takagi *et al.* reported that an extremely high salinity of 1.5 M markedly decreases the cell growth of *Dunaliella* sp. [14]. However, our results show that the lipid content increased significantly with the increase in sea salt concentration in the stationary phase. This indicates that high lipid content might not only come from salt stress but also from nitrogen limitation. Therefore, as observed by Pal *et al.*, combining more than one environmental stress (such as simultaneous high salinity and nitrogen depletion) may further enhance lipid accumulation in microalgae [31]. As shown in Table 3, after a seven-day nitrogen depletion, the highest lipid content resulting from cultivation at respective sea salt concentrations of 0.5, 2.0, 3.5, and 5.0% were 38.7, 53.5, 55.1, and 64.0%, respectively. In particular, through combining the stress of 5% sea salt and nitrogen limitation, the lipid content increased markedly from 14.5 to 64.0%, which is approximately a 4.4-fold enhancement. Unfortunately, the cell growth rate under high salinity could not be maintained as well as under low salinity, resulting in lower lipid productivity (Table 3). Thus, in order to enhance the potential of producing lipids from strain JSC4, the lipid productivity should be significantly improved by appropriate engineering strategies.

**Table 3:** **Combined effect of salinity and nitrogen limitation on biomass production, lipid accumulation, and CO**_**2 **_**fixation of *Chlamydomonas *sp. JSC4**

**Sea salt concentration (%)**	**Cultivation time under nitrogen depletion (days)**	**Biomass concentration (g L**^**-1**^**)**	**Lipid content (%)**	**Lipid productivity (mg L**^**-1 **^**d**^**-1**^**)**	**CO**_**2 **_**fixation rate (mg L**^**-1 **^**d**^**-1**^**)**
0.5	0	1.414 ± 0.082	15.6 ± 0.6	109 ± 3	1312 ± 81
	3	2.353 ± 0.078	29.0 ± 1.1	116 ± 0	755 ± 26
	5	2.691 ± 0.001	32.0 ± 0.3	107 ± 1	628 ± 0
	7	2.748 ± 0.011	38.7 ± 2.1	104 ± 5	507 ± 2
2.0	0	1.426 ± 0.008	15.8 ± 0.1	111 ± 0	1319 ± 6
	3	2.232 ± 0.007	41.9 ± 1.8	159 ± 8	713 ± 3
	5	2.467 ± 0.064	49.8 ± 2.3	152 ± 11	573 ± 16
	7	2.488 ± 0.044	53.5 ± 0.8	130 ± 4	457 ± 9
3.5	0	1.482 ± 0.029	15.3 ± 2.5	79 ± 119	968 ± 17
	3	2.018 ± 0.052	45.0 ± 0.1	143 ± 8	609 ± 18
	5	2.191 ± 0.033	51.8 ± 2.2	136 ± 8	494 ± 9
	7	2.242 ± 0.064	55.1 ± 0.8	121 ± 2	411 ± 13
5.0	0	1.135 ± 0.031	14.5 ± 0.7	56 ± 16	699 ± 15
	3	1.817 ± 0.057	51.4 ± 2.0	149 ± 1	528 ± 5
	5	2.058 ± 0.048	59.8 ± 1.2	148 ± 5	448 ± 10
	7	2.076 ± 0.033	64.0 ± 1.7	129 ± 6	371 ± 5

The values are the mean ± standard deviation of three replicated experiments. (Light intensity = 200 μmol m^-2^ s^-1^; CO_2_ aeration = 2.0%; CO_2_ flow rate = 0.05 vvm).

**Figure 4:**
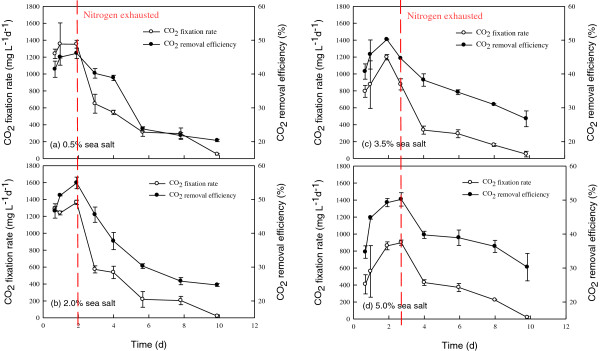
**Time course profiles of CO**_**2 **_**fixation rate and CO**_**2 **_**fixation ability during growth of *Chlamydomonas *sp. JSC4 on Modified Bold 3 N medium under the sea salt concentration of (a) 0.5%, (b) 2.0%, (c) 3.5%, and (d) 5.0%.** Error bars indicate standard deviation of three replicated experiments. (Light intensity = 200 μmol m^-2^ s^-1^; CO_2_ aeration = 2.0%; CO_2_ flow rate = 0.05 vvm).

Figure 4 depicts the time course of CO_2_ capture of the JSC4 strain under different salinities. The trends in CO_2_ fixation rate and CO_2_ removal efficiency under different sea salt concentrations were very similar throughout the course of the experiments, revealing low values at early and late cultivation periods, while reaching the highest values after cultivation for between two and three days. Similar trends were observed in our previous study [33], and the relationship between cell growth and CO_2_ fixation can be represented as a bell-shape curve. During the optimal time period indicated in Figure 4 and Table 3, the maximum CO_2_ removal efficiency and CO_2_ fixation rate (obtained with 2% sea salt) were 54.9% and 1319.0 mg L^-1^d^-1^, respectively, which were values superior to most of the relevant studies [34]. This excellent performance in CO_2_ fixation makes strain JSC4 a candidate for the practical CO_2_ fixation of industrial flue gas. It should be noted that the high CO_2_ fixation rate occurs when the microalgae grow very fast. During this fast growth period, lipid accumulation is usually insignificant (Figure 4 and Table 3). Therefore, normally a follow-up lipid accumulation operation (such as nitrogen depletion) should be conducted for the cells obtained from CO_2_ fixation to ensure high lipid production. However, as shown in Table 3, the highest lipid productivity of 158.9 mg L^-1^d^-1^ was obtained when the CO_2_ fixation rate was 712.9 mg L^-1^ d^-1^ (with 2% sea salt), a value superior to the performance of most other green microalgae under batch cultivation systems [34].

### Metabolic profiling of *Chlamydomonas* sp. JSC4 under salt stress and nitrogen depletion

**Figure 5:**
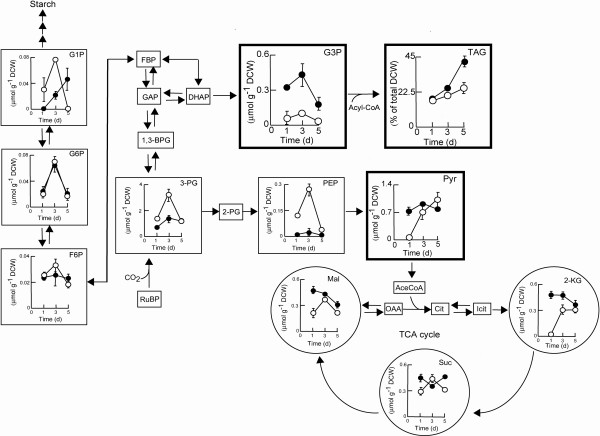
**Time course analysis of metabolite content of *Chlamydomonas *sp. JSC4 cells cultivated on Modified Bold 3 N medium with (closed circles) or without (open circles) 2% sea salt.** Error bars indicate standard deviation (n = 3).

To investigate the metabolic pathways of interest for lipid production, metabolite profiling appears to be a well suited method to monitor numerous changes of metabolite levels in response to various environmental stimuli [35]. However, the metabolic pathways involved in storage lipid accumulation in microalgae are poorly studied compared to higher plants [23]. Several environmental stresses, including nitrogen depletion and high salinity, have been used to increase the production of microalgal lipid [7]. However, little is known about the mechanism of lipid synthesis in microalgae under salt stress. To the best of our knowledge, this is the first report following the metabolic profiles of microalgae cultivated under the combined stresses of salinity and nitrogen depletion. Intracellular metabolites were extracted and identified using capillary electrophoresis coupled with mass spectrometry (CE/MS) and variations in the amount of various metabolites over time were determined. Figure 5 clearly shows that under nitrogen sufficient conditions (on day one), the levels of most triglyceride synthesis-related metabolites (glycerol-3-phosphate (G3P)) under salt stress were significantly higher than those in freshwater cultures. It has been reported salt-adding would significantly cause the osmotic stress in the medium which results in a dramatic accumulation of glycerol in the microalga *Chlamydomonas reinhardtii*[36]. Moreover, it is known that G3P is an extremely important metabolic intermediate for the synthesis of glycerol in many microorganisms used as a precursor for triacylglyceride [37]. Therefore, this phenomenon suggests that a positive relationship between G3P formation and triacylglyceride synthesis in microalgae under osmotic stress caused from adding salts existed; suggesting osmotic stress could obviously trigger the formation of G3P and further promote the synthesis of triacylglyceride. In addition, several key metabolites involved in the Calvin cycle (fructose-6-phosphate, 3-phosphoglyceric acid, and phosphoenolpyruvic acid), or tricarboxylic acid cycle (2-ketoglutaric acid) exhibited a significant increase until reaching their highest value at day three, suggesting that nutrients such as nitrogen in the medium or inside the cells might be sufficient to maintain cell viability. On the other hand, after nitrogen depletion for three days (day five), the levels of most of these metabolites tended to decrease, mainly due to the storage of energy compounds in the form of triacylglycerides (Figure 5). Moreover, an obvious increase in glucose-1-phosphate (G1P) under salt stress was observed, indicating that under certain environmental stresses, strain JSC4 may also store a certain amount of starch. Other studies reported an increased amount of lipids in starchless mutants of *Chlorella pyrenoidosa*[38] and in *C. reinhardtii*[39], suggesting a competition between lipid and carbohydrate synthesis. Therefore, blocking the carbohydrate biosynthesis pathway of JSC4 has the potential to further boost triacylglyceride production.

Notably, cultivation under conditions of nitrogen depletion led to a dramatic accumulation of pyruvate in freshwater cultures. This may be caused by the markedly slower accumulation rate of intermediates (such as 2-ketoglutaric acid, succinate, and malate) in the tricarboxylic acid cycle, thus restricting lipid synthesis [40]. In contrast, the amount of pyruvate remained almost constant under salt stress, which is in good agreement with the efficient accumulation of lipid shown in Figure 3. Furthermore, large amounts of G3P appear at an earlier stage of nitrogen depletion under salt stress when compared with that in freshwater. Dulermo and Nicaud [41] reported that modification of the G3P shuttle in *Yarrow lipolytica* improved lipid accumulation. These findings indicate that triacylglyceride synthesis may be limited by the availability of G3P and pyruvate. Using CE/MS, the relevant metabolism of strain JSC4 could be successfully profiled, and the information obtained can be applied to future genetic engineering of the lipid synthetic pathway.

### Two-stage strategy to enhance lipid accumulation of *Chlamydomonas* sp. JSC4

Although strain JSC4 is a promising feedstock for biodiesel production, its lipid content and lipid productivity should be further improved via various engineering strategies. Batch operations under high salt stress indicated that nitrogen depletion raised the lipid content dramatically, but also caused a significant decrease in biomass production, which is unfavorable to overall lipid productivity. In contrast, lipid accumulation under a combination of nitrogen depletion and low salt stress was not satisfactory, even though cell growth was slightly better under these conditions, as shown in Table 3. To avoid the above problems a two-stage cultivation strategy has been widely reported to improve lipid production [5,42]; however, very few studies have focused on salt as a stimulant in a second stage to enhance lipid accumulation of microalgae [43]. In this strategy, a nutrient-rich medium with 2% sea salt was used in the first stage, allowing cell growth, for approximately two days. After over 95% of the nitrate was consumed the culture was transferred to a nitrogen limited medium amended with 3, 5, or 7% sea salt to enhance lipid accumulation.

**Figure 6:**
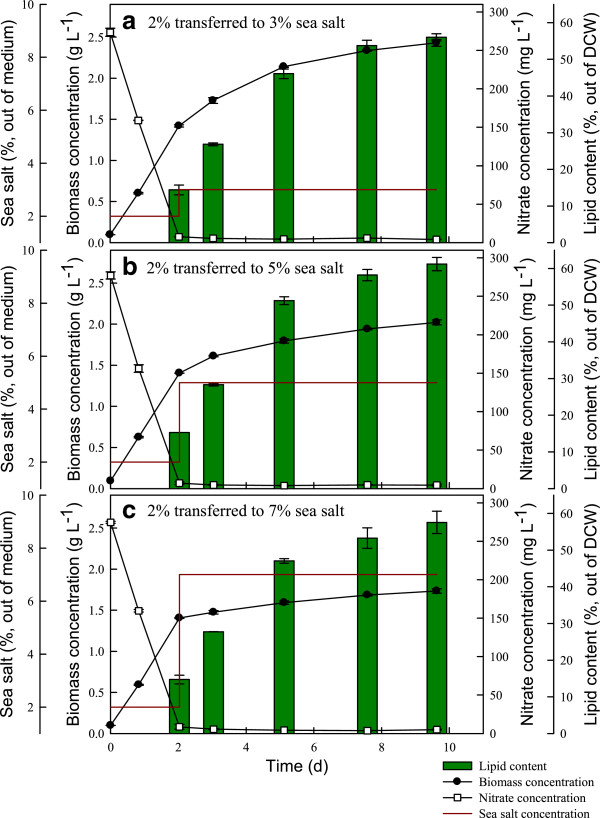
**Time course profiles of biomass concentration, nitrate concentration, and lipid content during the growth of *Chlamydomonas *sp. JSC4 on Modified Bold 3 N medium before and after transfer to high-salinity conditions of (a) 3%, (b) 5%, or (c) 7% sea salt.** Error bars indicate standard deviation of three replicated experiments. (Light intensity = 200 μmol m^-2^ s^-1^; CO_2_ aeration = 2.0%; CO_2_ flow rate = 0.05 vvm). DCW, dry cell weight.

As shown in Figure 6 and Table 4, after switching to the second stage for seven days, the lipid content of strain JSC4 in the presence of 3, 5, and 7% sea salt reached nearly 56, 61, and 58% (w/v), respectively, which is approximately four times higher than that obtained in the first stage (in which the lipid content was around 15%). Moreover, it was clear that the lipid content tended to increase slightly along with rising sea salt concentration, reaching the highest value of 61.2% in the presence of 5% sea salt. However, a further increase in sea salt concentration to 7% did not lead to a further increase in lipid content. Instead, it resulted in a slight drop in lipid content. This finding is consistent with a recent report showing that extremely high salinity strongly decreases lipid accumulation ability [44]. Thus, a two-stage cultivation strategy can significantly enhance the lipid content of strain JSC4, with a yield higher than that obtained in batch operation or in related studies (Table 4). However, the biomass productivity is markedly lowered by abruptly switching to a high salinity environment, probably as a consequence of poor cell growth. As a result, the highest lipid content (61.2%) was achieved when the second stage was performed at 5% sea salt, while the highest lipid productivity (183.9 mg L^-1^ d^-1^) was obtained when the second stage was performed at 3% sea salt. Therefore by properly adjusting the salinity in the second stage the optimal lipid production can be achieved.

**Table 4:** **Biomass concentration, lipid accumulation, and CO**_**2 **_**fixation of *Chlamydomonas *sp. JSC4 after transfer to high salinity conditions of 3, 5, and 7% sea salt for three to seven days**

**Final sea salt concentration (%)**	**Cultivation time under nitrogen depletion (days)**	**Biomass concentration (g L**^**-1**^**)**	**Lipid content (%)**	**Lipid productivity (mg L**^**-1 **^**d**^**-1**^**)**	**CO**_**2 **_**fixation rate (mg L**^**-1 **^**d**^**-1**^**)**
2.0 (batch)	3	2.232 ± 0.007	41.9 ± 1.8	159 ± 8	731 ± 3
	5	2.467 ± 0.064	49.8 ± 2.3	152 ± 11	561 ± 16
	7	2.488 ± 0.044	53.5 ± 0.8	130 ± 4	466 ± 9
3.0	3	2.141 ± 0.007	46.1 ± 1.3	184 ± 5	750 ± 3
	5	2.337 ± 0.009	53.8 ± 1.5	159 ± 5	556 ± 2
	7	2.431 ± 0.042	56.0 ± 1.0	136 ± 0	456 ± 8
5.0	3	1.796 ± 0.026	51.2 ± 1.1	170 ± 1	623 ± 10
	5	1.941 ± 0.010	58.2 ± 1.5	142 ± 4	458 ± 2
	7	2.022 ± 0.030	61.2 ± 1.8	122 ± 6	376 ± 6
7.0	3	1.593 ± 0.027	47.1 ± 0.6	138 ± 4	549 ± 8
	5	1.683 ± 0.013	53.3 ± 2.8	112 ± 7	394 ± 6
	7	1.735 ± 0.028	57.6 ± 3.0	98 ± 7	320 ± 5

Before transfer to high salinity, the cultures were grown with 2% sea salt for around two days. The values are the mean ± standard deviation of three replicated experiments. (Light intensity = 200 μmol m^-2^ s^-1^; CO_2_ aeration = 2.0%; CO_2_ flow rate = 0.05 vvm).

### Effect of stepwise addition of sea salt on cell growth and lipid accumulation of *Chlamydomonas* sp. JSC4

The results shown above indicate that cultivation of strain JSC4 under high salinity is likely to enhance the lipid content but lower the growth rate. To achieve higher lipid productivity, which is the major indicator of lipid production from an engineering aspect, the growth inhibition due to salinity stress should be alleviated. Several attempts have been made to achieve this goal. For example, Takagi *et al.* gradually increased the salt concentration to allow for the adaptation of microalgae to high salinity [14]. Therefore, using high salinity stress to stimulate lipid accumulation of microalgae and maintaining a satisfactory cell growth rate (for example, by enhancing adaptation to salinity) seems to be a promising strategy to enhance lipid content and lipid productivity simultaneously. This concept was realized in this study by means of salinity-gradient operations. Before employing the salinity gradient, the JSC4 strain was cultivated in batch mode until over 95% of the nitrogen source was consumed. At this point, the highest biomass productivity is approximately achieved. The operation was then switched to salinity-gradient mode by a stepwise increase in the sea salt concentration in increments of 0.5, 1.0, or 1.5% per day for five days. The cell growth, salinity level, residual nitrate concentration, CO_2_ fixation rate, and lipid content were monitored during salinity-gradient operations.

**Figure 7:**
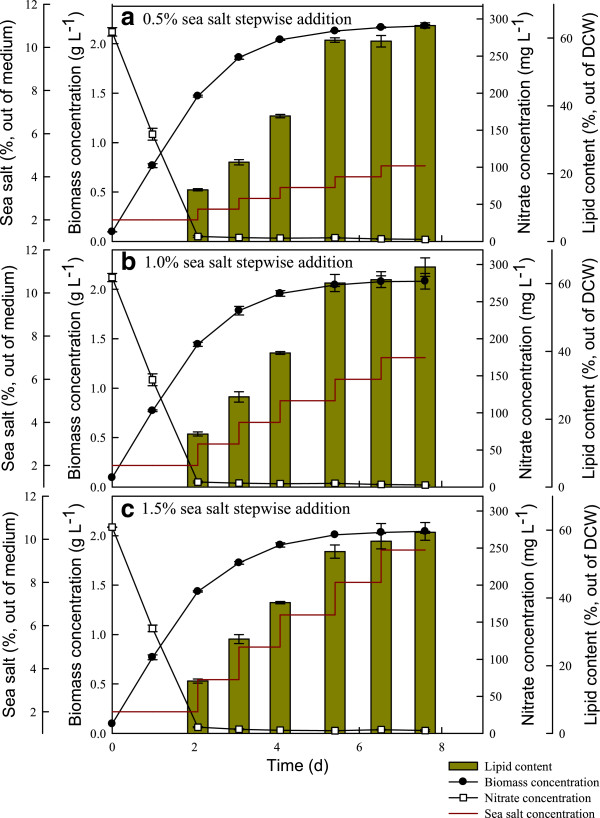
**Time course profiles of biomass concentration, nitrate concentration, and lipid content during growth of *Chlamydomonas *sp. JSC4 before and after gradient operation with (a) 0.5%, (b) 1.0%, or (c) 1.5% sea salt.** Error bars indicate standard deviation of three replicated experiments. (Light intensity = 200 μmol m^-2^ s^-1^; CO_2_ aeration = 2.0%; CO_2_ flow rate = 0.05 vvm). DCW, dry cell weight.

In Figure 7 and Table 5, it is clear that the stepwise addition of sea salt rapidly enhanced the lipid content without significant inhibition of cell growth. This resulted in higher lipid productivity, especially for the treatment supplying a smaller amount of sea salt (an increment of 0.5% sea salt per day). The maximum lipid productivity (223.2 mg L^-1^d^-1^) and lipid content (59.4%) were obtained when the salinity gradient was operated at an increment of 0.5% per day. This performance is much higher than that obtained from our batch system (158.9 mg L^-1^d^-1^) or two-stage operation system (183.9 mg L^-1^d^-1^). The maximum lipid productivity obtained from optimal salinity-gradient operation was also significantly higher than that obtained from most related studies (in the range of 51.0 to 140.8 mg L^-1^d^-1^; Table 6).

**Table 5:** **Performance based on biomass concentration, lipid accumulation, and CO**_**2 **_**fixation of salinity-gradient culturing of *Chlamydomonas *sp. JSC4 with stepwise addition of sea salt at different increments**

**Sea salt addition (% d**^**-1**^**)**	**Cultivation time under nitrogen depletion (days)**	**Biomass concentration (g L**^**-1**^**)**	**Lipid content (%)**	**Lipid productivity (mg L**^**-1 **^**d**^**-1**^**)**	**CO**_**2 **_**fixation rate (mg L**^**-1 **^**d**^**-1**^**)**
0.0 (batch)	0	1.426 ± 0.008	15.8 ± 0.1	111 ± 0	1319 ± 6
	3	2.232 ± 0.007	41.9 ± 1.8	159 ± 8	731 ± 3
	5	2.467 ± 0.064	49.8 ± 2.3	152 ± 11	561 ± 16
0.5	0	1.471 ± 0.010	15.3 ± 0.3	101 ± 3	1243 ± 9
	3	2.129 ± 0.004	59.4 ± 0.7	223 ± 2	706 ± 1
	5	2.180 ± 0.042	63.7 ± 0.8	175 ± 3	515 ± 3
1.0	0	1.445 ± 0.021	15.7 ± 0.6	102 ± 5	1219 ± 19
	3	2.044 ± 0.015	60.2 ± 2.5	217 ± 7	677 ± 5
	5	2.082 ± 0.079	64.9 ± 2.7	170 ± 14	491 ± 20
1.5	0	1.436 ± 0.007	15.5 ± 0.6	100 ± 3	1211 ± 6
	3	2.009 ± 0.001	53.7 ± 2.0	190 ± 7	665 ± 0
	5	2.044 ± 0.090	59.4 ± 0.3	152 ± 6	482 ± 22

The values are the mean ± standard deviation of three replicated experiments. (Light intensity = 200 μmol m^-2^ s^-1^; CO_2_ aeration = 2.0%; CO_2_ flow rate = 0.05 vvm).

**Table 6:** **Comparison of lipid content and lipid productivity in *Chlamydomonas *sp. JSC4 and other marine microalgae reported in the literature**

** Species**	**Cultivation system**	**Lipid content (%)**	**Lipid productivity (mg L**^**-1 **^**d**^**-1**^**)**	**Reference**
*Chlorella sorokiniana* CY1	Semi-batch	57.7	140.8	[12]
*Chlorella vulgaris*	Batch	17.3	79.1	[26]
*Chlorella emersonii*	Batch	18.6	54.4	[26]
*Nannochloropsis* sp.	Batch	31.3	N.A	[45]
*Nannochloropsis* sp.	Two-stage	59.9	74.2	[11]
*Nannochloropsis* sp. F&M-M24	Semi-continuous	68.5	110.1	[13]
*Tetraselmis suecica* F&M-M33	Semi-continuous	39.1	53.1	[13]
*D. tertiolecta* UTEX LB999	Batch	25.9	N.A.	[46]
*T. pseudonana* CCMP1335	Batch	20.3	N.A.	[46]
*Isochrysis zhangjiangensis*	Two-stage	46.0	136.2	[16]
*Nannochloropsis gaditana*	Two-stage	73.1	51	[10]
*D. tertiolecta* ATCC30929	Batch	67.0	N.A.	[14]
*Chlorella protothecoides*	Batch	43.4	N.A	[15]
*Nannochloropsis* sp. F&M-M28	Batch	35.7	60.9	[47]
*Nannochloropsis* sp. F&M-M26	Batch	29.6	61.0	[47]
***Chlamydomonas *sp. JSC4**	**Batch**	**41.9**	**159**	**This study**
***Chlamydomonas *sp. JSC4**	**Two-stage**	**46.1**	**184**	**This study**
***Chlamydomonas *sp. JSC4**	**Gradient**	**59.4**	**223**	**This study**

N.A.: not available.

To date, no studies have investigated a gradient operation strategy utilizing the salinity effect for the enhancement of lipid content and lipid productivity and only a few reports have mentioned that salinity adjustment in a batch system can increase lipid accumulation [14,31,44,48]. This study demonstrated that the combined effect of high salinity stress and nitrogen depletion can indeed increase the lipid production performance and that an increasing gradient of salinity led to a much higher lipid productivity of 223.2 mg L^-1^ d^-1^ compared to conventional batch and two-stage operations. Moreover, as indicated in Table 7, the FAs resulting from the salinity-gradient operation was mainly composed of saturated and monounsaturated FAs, which accounted for approximately 60% of the total fatty acid methyl esters (FAMEs). This lipid composition, which is similar to that obtained in batch and two-stage cultivation systems, is suitable for biodiesel production. Therefore, strain JSC4 cultivated under optimal salinity-gradient operation appears to provide both high lipid productivity and good lipid quality. Therefore, in addition to the conventional engineering strategies used for stimulating lipid accumulation, such as nutrient deficiency, this study offers a new way to increase the lipid productivity of microalgae using a fairly simple but effective method.

**Table 7:** **FA composition of *Chlamydomonas *sp. JSC4 cultured under batch, two-stage, or salinity-gradient operation**

	***Chlamydomonas *sp. JSC4 FA composition (%)**		
	**Batch**	**Two-stage**	**Salinity gradient**
Palmitic acid (C16:0)	27.6 ± 0.3	30.7 ± 0.8	30.7 ± 0.5
Palmitoleic acid (C16:1)	3.2 ± 0.1	2.9 ± 0.1	2.7 ± 0.2
Stearic acid (C18:0)	3.1 ± 0.1	2.8 ± 0.2	2.6 ± 0.2
Oleic acid (C18:1)	26.6 ± 0.6	23.4 ± 0.1	22.8 ± 1.1
Linoleic acid (C18:2)	25.3 ± 0.3	25.1 ± 0.1	25.0 ± 0.3
Linolenic acid (C18:3)	5.4 ± 0.1	5.0 ± 0.1	5.4 ± 0.4
C16 & C18 groups	91.2 ± 1.3	89.9 ± 0.8	89.2 ± 0.4
Lipid content (%)	41.9 ± 1.8	46.1 ± 1.3	59.4 ± 0.7
Lipid productivity (mg L^-1^ d^-1^)	158.9 ± 7.6	183.9 ± 4.6	223.2 ± 2.1

The values are the mean ± standard deviation of three replicated experiments. (Light intensity = 200 μmol m^-2^ s^-1^; CO_2_ aeration = 2.0%; CO_2_ flow rate = 0.05 vvm).

## Conclusions

This work integrates metabolic profiling and biochemical engineering approaches to demonstrate a feasible and effective technology able to greatly enhance the lipid productivity of the marine microalga *Chlamydomonas* sp. JSC4. The information obtained from metabolic profiles suggested that levels of the key metabolite G3P are very high under salt stress, which could be why lipid accumulation is promoted. Moreover, enhancement of G1P levels under salt stress was observed with competition between lipid and carbohydrate synthesis. The engineering studies showed that by appropriately combining the stresses of salinity and nitrogen depletion, lipid accumulation of the microalgal strain can be significantly improved. In particular, application of an innovative salinity gradient, when combined with nitrogen depletions, could significantly increase the lipid content and lipid productivity of *strain* JSC4 to 59.4% and 223.2 mg L^-1^d^-1^, respectively, which are higher than most previously reported values for both freshwater and seawater microalgal strains. The lipids produced by strain JSC4 were mainly composed of saturated and monounsaturated fatty acids, which are very suitable for biodiesel production.

## Materials and methods

### Microalgal strain and medium for pre-culturing

The microalga used in this study was *Chlamydomonas* sp. JSC4, which was isolated from a coastal area of southern Taiwan. The 18S rDNA sequence of this strain has been deposited in the NCBI GenBank database (with an accession number of [KF3:83270]). The medium used for its pre-culture was Modified Bold 3 N medium (4.4 mM NaNO_3_, 0.22 mM K_2_HPO_4_, 0.3 mM MgSO_4_ · 7H_2_O, 0.17 mM CaCl_2_ · 2H_2_O, 0.43 mM KH_2_PO_4_, 0.43 mM NaCl; the levels of metals in the medium were the same as those described in Berges *et al.*[49]). The microalga was routinely grown at between 26 to 28°C for five to six days with a continuous supply of 2.0% CO_2_ at an aeration rate of 0.05 vvm. The culture was illuminated continuously with a light intensity of approximately 150 μmol m^-2^ s^-1^. The light intensity was measured by a Li-250 Light Meter with a Li-190SA pyranometer sensor (Li-COR Inc., Lincoln, Nebraska, United States).

### Operation of photobioreactor (PBR)

The PBR used in this study was a 1 L glass vessel (15.5 cm long and 9.5 cm in diameter) equipped with external light sources (DURAN, Mainz, Germany). The microalga was grown in the PBR on five different media; namely, Modified Bold 3 N medium [49], Modified Bristol 1/2 N medium [18], BG11 1/4 N medium [50], and basal medium 1/3 N [18]. The JSC4 strain was pre-cultured and inoculated into the PBR with an inoculum size of 90 to 100 mg L^-1^. For batch cultures, the microalga was cultivated at 27 to 28°C, pH 6.5 to 7.5, at an agitation rate of 300 rpm under a light intensity of approximately 200 μmol m^-2^ s^-1^. As the sole carbon source, 2.0% CO_2_ was fed into the microalgal culture continuously at an aeration rate of 0.05 vvm. Liquid samples were collected from the sealed glass vessel at designated time intervals to determine the microalgal cell concentration, pH, and residual nitrate concentration. The amount of CO_2_ reduction was determined by measuring the difference between the CO_2_ concentrations in the influent and effluent streams of the PBR via a GM70 CO_2_ detector (Vaisala, Tokyo, Japan).

### Two-stage cultivation

The two-stage cultivation system was set up in a single PBR containing a 1 L microalgal culture. The operation was started with a batch culture at 2% sea salt (Sigma-Aldrich Co., St. Louis, United States) and 2.0% CO_2_ aeration at a feeding rate of 0.05 vvm. When the nitrogen consumption was over 95%, different amounts of sea salt were added to the broth until it reached a sea salt concentration of 3, 5, or 7%. Liquid samples were collected from the sealed glass vessel at designated time intervals to determine the microalgal cell concentration, pH, and residual nitrate concentration.

### Salinity-gradient cultivation

The stepwise salinity-gradient cultivation system was set up in a single PBR containing a 1 L microalgal culture. The operation was started with a batch culture at 2% sea salt and 2.0% CO_2_ aeration at a feeding rate of 0.05 vvm. When the nitrogen consumption was over 95%, the system was switched to stepwise mode with an incremental increase in the sea salt level of 0.5, 1.0, or 1.5% per day for five days. As the sole carbon source, 2.0% CO_2_ was fed into the culture continuously at 0.05 vvm during the course of the stepwise culture. Liquid samples were collected from the sealed glass vessel at various sea salt addition intervals to determine the microalgal cell concentration, pH, and residual nitrate concentration.

### Determination of microalgal cell concentration

The microalgal cell concentration in the PBR was regularly determined by measuring the optical density at a wavelength of 682 nm (denoted as OD_682_) using a UVmini-1240 UV/Vis spectrophotometer (Shimadzu, Kyoto, Japan) after proper dilution with deionized water (EMD Millipore, Darmstadt, Germany) to give a range of measurement between 0.1 and 0.9. The dry cell weight (DCW) of the microalgal biomass was obtained by filtering 50 mL aliquots of culture through a cellulose acetate membrane filter (0.45 μm pore size, 47 mm diameter) (EMD Millipore, Darmstadt, Germany). Each loaded filter was freeze-dried until the weight was invariant. The dry weight of the blank filter was subtracted from that of the loaded filter to obtain the microalgal DCW. The OD_682_ values were also converted to biomass concentration via appropriate calibration between OD_682_ and DCW, and the conversion factor was determined as 1.0 OD_682_ equaling approximately 0.7 to 0.8 g DCW L^-1^.

### Measurement of residual nitrate content

Nitrate concentration was determined according to the modified method reported in our previous study [20]. Each sample collected from the PBR was filtered with a 0.22 μm pore size filter and then diluted 20-fold with deionized water. The residual nitrate content of the diluted samples was determined according to the optical density measured at a wavelength of 220 nm (OD_220_) with appropriate calibrations. The conversion factor was determined as 1.0 OD_220_ equaling approximately 389.3 mg nitrate L^-1^.

### Determination of growth kinetic parameters and CO_2_ fixation performance

A time course of the biomass concentration (mg L^-1^) was used to calculate the specific growth rate (d^-1^) based on a DCW (on a logarithmic scale) versus time plot. The biomass productivity (mg L^-1^ d^-1^) was calculated as follows:

(1) $$P=\frac{\mathrm{\Delta X}}{\mathrm{\Delta t}}$$

where ΔX is the variation in biomass concentration (mg L^-1^) within a cultivation time of Δt (d).

Moreover, the CO_2_ fixation rate (*P*_CO2_; mg L^-1^d^-1^) was calculated as follows:

(2) $$P_{{\mathrm{CO}}_{2}}\left(\mathrm{mg}/L/d\right)=1.88\times P_{\mathrm{biomass}}$$

where *P*_biomass_ is the biomass productivity (mg L^-1^d^-1^) described above. The typical molecular formula of microalgal biomass, CO_0.48_H_1.83_N_0.11_P_0.01_, [8] was used in this study.

The CO_2_ fixation efficiency (%) was determined as follows:

(3) $$\begin{array}{l} {\mathrm{CO}}_{2}\;\mathrm{fixation}\;\mathrm{efficiency}\left(\%\right)\\ \;=\;\frac{C_{\mathrm{CO}2,\;\mathrm{influent}}\;-\;C_{\mathrm{CO}2,\;\mathrm{effluent}}}{{\;C}_{\mathrm{CO}2,\;\mathrm{influent}}}\times 100 \end{array}$$

where C_CO2,influent_ and C_CO2,effluent_ are the concentrations of CO_2_ in the influent and effluent streams, respectively.

### Determination of the lipid content and fatty acid profile

After allowing an appropriate time for nitrogen consumption (zero to seven days of nitrogen depletion), the microalgal cells were harvested from the culture medium by centrifugation (7000 rpm for 2 minutes). The cells were washed twice with deionized water, lyophilized, and weighed. The lipid composition was determined as FAMEs following direct transesterification of lipids using the method described in Ho *et al.*[19]. The FAMEs were analyzed by gas chromatography-mass spectrometry (GC/MS) on a GCMS-QP2010 Plus instrument (Shimadzu, Kyoto, Japan). Samples were injected into a DB-23 capillary column (60 m, 0.25 mm internal diameter, 0.15 μm film thickness; Agilent Technologies, Palo Alto, California, United States). Helium was used as the carrier gas with a flow rate of 2.3 mL min^-1^. The temperatures of the injector, ion source, and interface source were set at 230, 230, and 250°C, respectively. The oven temperature was initially set at 50°C for 1 minute, increasing from 50 to 175°C at a heating rate of 25°C/min, then from 175 to 230°C at a heating rate of 4°C per minute, and held at 230°C for 5 minutes. The purified FAMEs were identified based on retention time and the pattern of fragmentation by electron impact analysis. Supelco 37 Component FAME Mix (Sigma-Aldrich Co., St. Louis, Missouri, United States) was utilized as a quantitative standard and pentadecanoic acid (Sigma-Aldrich Co., St. Louis, United States) was used as an internal standard.

### Sampling procedures for metabolic profile analysis

Cell sampling was performed according to our previously reported method [22] with minor modifications. Microalgal cells, equivalent to 5 to 10 mg dry weight, were removed from cultivation vessels and added into 4-fold volume of pre-chilled quenching solution (–30°C) which was Modified Bold 3 N medium containing 32.5% methanol, then filtered using 1 μm pore size Omnipore filter disks (Millipore, Massachusetts, United States). After washing with 20 mM ammonium bicarbonate pre-chilled to 4°C, cells retained on the filters were immediately placed into 1 mL precooled (–30°C) methanol containing 12.4 μM piperazine-1,4-bis(2-ethanesulfonic acid) as the internal standard for mass analysis. Intracellular metabolites were extracted using a cold 10:3:1 (v/v/v) methanol: chloroform: water solution, as described previously [35]. Cells were suspended by vortexing and then 1 mL of the cell suspension was mixed with 100 μL precooled (4°C) water and 300 μL chloroform. The cell suspension was shaken at 1200 rpm in a model MBR-022UP incubator (TAITEC, Saitama, Japan) for 30 minutes at 4°C in the dark before centrifugation at 14 000 *g* for 5 minutes at 4°C. Next, 980 μL of the cell extract obtained as the supernatant was transferred to a clean tube. After adding 440 μL water, aqueous and organic layers were phase-separated by centrifugation at 14 000 *g* for 5 minutes at 4°C. After filtration through a Millipore 5 kDa cut-off filter (Millipore, Massachusetts, United States) for the removal of solubilized proteins, the aqueous-layer extracts were evaporated under vacuum using a FreeZone 2.5 Plus freeze dry system (Labconco, Kansas City, Missouri, United States). Dried extracts were stored at –80°C until analysis by CE/MS.

### Capillary electrophoresis/mass spectrometry metabolite analysis

The metabolites were determined through the CE/MS method described in Hasunuma *et al.*[22]. Dried metabolites were dissolved in 20 μL Milli-Q water (EMD Millipore, Darmstadt, Germany) before analysis. The CE/MS experiments were performed using an Agilent G7100 CE system, an Agilent G6224AA LC/MSD time-of-flight (TOF) system (Agilent, Santa Clara, United States), and an Agilent 1200 series isocratic HPLC pump (Agilent, Santa Clara, United States) equipped with a 1:100 splitter for delivery of the sheath liquid.

Agilent ChemStation software (Agilent, Santa Clara, United States) for CE and MassHunter software (Agilent, Santa Clara, United States) for the Agilent TOFMS system were used for system control and data acquisition, respectively. The analytical conditions for anionic metabolite analyses were as described previously [22]. The CE separations were performed in a fused silica capillary (1 m × 50 μm i.d.) filled with 50 mM ammonium acetate (pH 9) for anionic metabolite analyses. The CE polarity was such that the electrolyte vial (inlet) was at the anode, and the electrospray ionization (ESI) probe (outlet) was at the cathode. Samples were injected into the CE system at a pressure of 50 mbar for 30 seconds. The voltage applied to the CE capillary was set at 30 kV, with a ramp time of 0.3 minutes. For anionic metabolite analyses the electrolyte was passed through the capillary using an air pump and was delivered at a pressure of 10 mbar from 0.4 to 30 minutes and 100 mbar from 30.1 to 49.5 minutes. The flow rate of the sheath liquid was set at 8 μL min^-1^. The ESI-MS analyses were conducted in either the positive or negative ion mode using a capillary voltage of –3.5 or 3.5 kV, respectively. The TOF-MS fragmenter, skimmer, and Oct RFV were set to 100, 65, and 750 V, respectively. The flow of heated drying nitrogen gas (300°C) was maintained at 10 L min^-1^. Mass data were acquired at a rate of 1 spectrum s^-1^ over the mass-to-charge ration (*m/z*) range of 70 to 1000 m/z.

## Abbreviations

2-KG: 2-ketoglutarate; 2-PG: 2-phosphoglycerate; 3-PG: 3-phosphoglycerate; AceCoA: Acetyl-CoA; CE/MS: Capillary electrophoresis-mass spectrometry; Cit: Citrate; DHAP: Dihydroxyacetone phosphate; DCW: Dry cell weight; ESI: Electrospray ionization; F6P: Fructose-6-phosphate; FA: Fatty acid; FAMEs: Fatty acid methyl esters; FBP: Fructose-1,6-bisphosphate; G1P: Glucose-1-phosphate; G3P: Glycerol-3-phosphate; G6P: Glucose-6-phosphate; GAP: Glyceraldehyde-3-phosphate; GC/MS: Gas chromatography-mass spectrometry; Icit: Isocitrate; Mal: Malate; NCBI: National Center for Biotechnology Information; OAA: Oxaloacetate; OD: Optical density; PBR: Photobioreactor; PEP: Phosphoenolpyruvate; Pyr: Pyruvate; RuBP: Ribulose-1,5-bisphosphate; Suc: Succinate; TAG: Triacylglycerol; TOF: Time-of-flight.

## Competing interests

The authors declare that they have no competing interests.

## Authors’ contributions

SHH performed and operated all experiments and drafted the manuscript. AN assisted to perform and analyze the metabolic experiments. XY contributed to the manuscript draft. JSC served as critical reviewer of the manuscript. KH contributed to advice the content of the manuscript. TH and AK coordinated the study and helped in drafting the manuscript. All authors read and approved the final manuscript.

## References

[B1] Sivakumar G Vail DR Xu J Burner DM Lay JO Ge X Weathers PJ Bioethanol and biodiesel: Alternative liquid fuels for future generations Eng Life Sci 2010 10 8 18

[B2] Ho S-H Huang S-W Chen C-Y Hasunuma T Kondo A Chang J-S Characterization and optimization of carbohydrate production from an indigenous microalga *Chlorella vulgaris* FSP-E Bioresour Technol 2013 135 157 165 23186680 10.1016/j.biortech.2012.10.100

[B3] Yen H-W Hu IC Chen C-Y Ho S-H Lee D-J Chang J-S Microalgae-based biorefinery – from biofuels to natural products Bioresour Technol 2013 135 166 174 23206809 10.1016/j.biortech.2012.10.099

[B4] Ho S-H Huang S-W Chen C-Y Hasunuma T Kondo A Chang J-S Bioethanol production using carbohydrate-rich microalgae biomass as feedstock Bioresour Technol 2013 135 191 198 23116819 10.1016/j.biortech.2012.10.015

[B5] Ho S-H Chen W-M Chang J-S *Scenedesmus obliquus* CNW-N as a potential candidate for CO_2_ mitigation and biodiesel production Bioresour Technol 2010 101 8725 8730 20630743 10.1016/j.biortech.2010.06.112

[B6] Liu ZY Wang GC Zhou BC Effect of iron on growth and lipid accumulation in *Chlorella vulgaris* Bioresour Technol 2008 99 4717 4722 17993270 10.1016/j.biortech.2007.09.073

[B7] Chen C-Y Yeh K-L Aisyah R Lee D-J Chang J-S Cultivation, photobioreactor design and harvesting of microalgae for biodiesel production: a critical review Bioresour Technol 2011 102 71 81 20674344 10.1016/j.biortech.2010.06.159

[B8] Chisti Y Biodiesel from microalgae Biotechnol Adv 2007 25 294 306 17350212 10.1016/j.biotechadv.2007.02.001

[B9] Gouveia L Marques AE da Silva TL Reis A *Neochloris oleabundans* UTEX#1185: a suitable renewable lipid source for biofuel production J Ind Microbiol Biotechnol 2009 36 821 826 19377896 10.1007/s10295-009-0559-2

[B10] San Pedro A González-López CV Acién FG Molina-Grima E Marine microalgae selection and culture conditions optimization for biodiesel production Bioresour Technol 2013 134 353 361 23524159 10.1016/j.biortech.2013.02.032

[B11] Jiang L Luo S Fan X Yang Z Guo R Biomass and lipid production of marine microalgae using municipal wastewater and high concentration of CO_2_ Appl Energ 2011 88 3336 3341

[B12] Chen C-Y Chang J-S Chang H-Y Chen T-Y Wu J-H Lee W-L Enhancing microalgal oil/lipid production from *Chlorella sorokiniana* CY1 using deep-sea water supplemented cultivation medium Biochem Eng J 2013 77 74 81

[B13] Bondioli P Della Bella L Rivolta G Chini Zittelli G Bassi N Rodolfi L Casini D Prussi M Chiaramonti D Tredici MR Oil production by the marine microalgae *Nannochloropsis* sp. F&amp;M-M24 and *Tetraselmis suecica* F&amp;M-M33 Bioresour Technol 2012 114 567 572 22459965 10.1016/j.biortech.2012.02.123

[B14] Takagi M Karseno S Yoshida T Effect of salt concentration on intracellular accumulation of lipids and triacylglyceride in marine microalgae *Dunaliella* cells J Biosci Bioeng 2006 101 223 226 16716922 10.1263/jbb.101.223

[B15] Campenni L Nobre BP Santos CA Oliveira A Aires-Barros M Palavra A Gouveia L Carotenoid and lipid production by the autotrophic microalga *Chlorella protothecoides* under nutritional, salinity, and luminosity stress conditions Appl Microbiol Biotechnol 2013 97 1383 1393 23160982 10.1007/s00253-012-4570-6

[B16] Feng D Chen Z Xue S Zhang W Increased lipid production of the marine oleaginous microalgae *Isochrysis zhangjiangensis* (Chrysophyta) by nitrogen supplement Bioresour Technol 2011 102 6710 6716 21524571 10.1016/j.biortech.2011.04.006

[B17] Williams PJB Laurens LM Microalgae as biodiesel & biomass feedstocks: Review & analysis of the biochemistry, energetics & economics Energ Environ Sci 2010 3 554 590

[B18] Yeh K-L Chang J-S Effects of cultivation conditions and media composition on cell growth and lipid productivity of indigenous microalga *Chlorella vulgaris* ESP-31 Bioresour Technol 2012 105 120 127 22189073 10.1016/j.biortech.2011.11.103

[B19] Ho S-H Chen C-Y Chang J-S Effect of light intensity and nitrogen starvation on CO_2_ fixation and lipid/carbohydrate production of an indigenous microalga *Scenedesmus obliquus* CNW-N Bioresour Technol 2012 113 244 252 22209130 10.1016/j.biortech.2011.11.133

[B20] Ho S-H Kondo A Hasunuma T Chang J-S Engineering strategies for improving the CO_2_ fixation and carbohydrate productivity of *Scenedesmus obliquus* CNW-N used for bioethanol fermentation Bioresour Technol 2013 143 163 171 23792755 10.1016/j.biortech.2013.05.043

[B21] Baran R Reindl W Northen TR Mass spectrometry based metabolomics and enzymatic assays for functional genomics Curr Opin Microbiol 2009 12 547 552 19695948 10.1016/j.mib.2009.07.004

[B22] Hasunuma T Kikuyama F Matsuda M Aikawa S Izumi Y Kondo A Dynamic metabolic profiling of cyanobacterial glycogen biosynthesis under conditions of nitrate depletion J Exp Bot 2013 64 2943 2954 23658429 10.1093/jxb/ert134 PMC3697948

[B23] Hu Q Sommerfeld M Jarvis E Ghirardi M Posewitz M Seibert M Darzins A Microalgal triacylglycerols as feedstocks for biofuel production: perspectives and advances Plant J 2008 54 621 639 18476868 10.1111/j.1365-313X.2008.03492.x

[B24] Siaut M Cuiné S Cagnon C Fessler B Nguyen M Carrier P Beyly A Beisson F Triantaphylidès C Li-Beisson Y Gilles P Oil accumulat ion in the model green alga *Chlamydomonas reinhardtii*: characterization, variability between common laboratory strains and relationship with starch reserves BMC Biotechnol 2011 11 7 21255402 10.1186/1472-6750-11-7 PMC3036615

[B25] Radakovits R Jinkerson RE Darzins A Posewitz MC Genetic engineering of algae for enhanced biofuel production Eukaryot Cell 2010 9 486 501 20139239 10.1128/EC.00364-09 PMC2863401

[B26] Talebi AF Mohtashami SK Tabatabaei M Tohidfar M Bagheri A Zeinalabedini M Hadavand Mirzaei H Mirzajanzadeh M Malekzadeh Shafaroudi S Bakhtiari S Fatty acids profiling: a selective criterion for screening microalgae strains for biodiesel production Algal Res 2013 2 258 267

[B27] Harwati TU Willke T Vorlop KD Characterization of the lipid accumulation in a tropical freshwater microalgae *Chlorococcum* sp Bioresour Technol 2012 121 54 60 22858468 10.1016/j.biortech.2012.06.098

[B28] Knothe G Analyzing biodiesel: standards and other methods J Am Oil Chem Soc 2006 83 823 833

[B29] Tang H Abunasser N Garcia MED Chen M Simon Ng KY Salley SO Potential of microalgae oil from *Dunaliella tertiolecta* as a feedstock for biodiesel Appl Energ 2011 88 3324 3330

[B30] Pérez Á Casas A Fernández CM Ramos MJ Rodríguez L Winterization of peanut biodiesel to improve the cold flow properties Bioresour Technol 2010 101 7375 7381 20547059 10.1016/j.biortech.2010.04.063

[B31] Pal D Khozin-Goldberg I Cohen Z Boussiba S The effect of light, salinity, and nitrogen availability on lipid production by *Nannochloropsis* sp Appl Microbiol Biotechnol 2011 90 1429 1441 21431397 10.1007/s00253-011-3170-1

[B32] Zhila N Kalacheva G Volova T Effect of salinity on the biochemical composition of the alga *Botryococcus braunii* Kütz IPPAS H-252 J Appl Phycol 2011 23 47 52

[B33] Ho S-H Lu W-B Chang J-S Photobioreactor strategies for improving the CO_2_ fixation efficiency of indigenous *Scenedesmus obliquus* CNW-N: statistical optimization of CO_2_ feeding, illumination, and operation mode Bioresour Technol 2012 105 106 113 22177736 10.1016/j.biortech.2011.11.091

[B34] Ho S-H Chen C-Y Lee D-J Chang J-S Perspectives on microalgal CO2-emission mitigation systems – a review Biotechnol Adv 2011 29 189 198 21094248 10.1016/j.biotechadv.2010.11.001

[B35] Bölling C Fiehn O Metabolite profiling of *Chlamydomonas reinhardtii* under nutrient deprivation Plant Physiol 2005 139 1995 2005 16306140 10.1104/pp.105.071589 PMC1310576

[B36] Wang Z Zhuge J Fang H Prior BA Glycerol production by microbial fermentation: a review Biotechnol Adv 2001 19 201 223 14538083 10.1016/s0734-9750(01)00060-x

[B37] Husic HD Tolbert N Effect of osmotic stress on carbon metabolism in *Chlamydomonas reinhardtii* accumulation of glycerol as an osmoregulatory solute Plant Physiol 1986 82 594 596 16665074 10.1104/pp.82.2.594 PMC1056165

[B38] Ramazanov A Ramazanov Z Isolation and characterization of a starchless mutant of *Chlorella pyrenoidosa* STL-PI with a high growth rate, and high protein and polyunsaturated fatty acid content Phycol Res 2006 54 255 259

[B39] Li Y Han D Hu G Sommerfeld M Hu Q Inhibition of starch synthesis results in overproduction of lipids in *Chlamydomonas reinhardtii* Biotechnol Bioeng 2010 107 258 268 20506159 10.1002/bit.22807

[B40] Ke J Behal RH Back SL Nikolau BJ Wurtele ES Oliver DJ The role of pyruvate dehydrogenase and acetyl-coenzyme A synthetase in fatty acid synthesis in developing *Arabidopsis* seeds Plant Physiol 2000 123 497 508 10859180 10.1104/pp.123.2.497 PMC59018

[B41] Dulermo T Nicaud J-M Involvement of the G3P shuttle and β-oxidation pathway in the control of TAG synthesis and lipid accumulation in *Yarrowia lipolytica* Metab Eng 2011 13 482 491 21620992 10.1016/j.ymben.2011.05.002

[B42] Su CH Chien LJ Gomes J Lin YS Yu YK Liou JS Syu RJ Factors affecting lipid accumulation by *Nannochloropsis oculata* in a two-stage cultivation process J Appl Phycol 2011 23 903 908

[B43] Xia L Ge H Zhou X Zhang D Hu C Photoautotrophic outdoor two-stage cultivation for oleaginous microalgae *Scenedesmus obtusus* XJ-15 Bioresour Technol 2013 144 261 267 23876654 10.1016/j.biortech.2013.06.112

[B44] Bartley ML Boeing WJ Corcoran AA Holguin FO Schaub T Effects of salinity on growth and lipid accumulation of biofuel microalga *Nannochloropsis salina* and invading organisms Biomass Bioenergy 2013 54 83 88

[B45] Wahidin S Idris A Shaleh SRM The influence of light intensity and photoperiod on the growth and lipid content of microalgae *Nannochloropsis* sp Bioresour Technol 2013 129 7 11 23232218 10.1016/j.biortech.2012.11.032

[B46] Jiang Y Yoshida T Quigg A Photosynthetic performance, lipid production and biomass composition in response to nitrogen limitation in marine microalgae Plant Physiol Biochem 2012 54 70 77 22387274 10.1016/j.plaphy.2012.02.012

[B47] Rodolfi L Chini Zittelli G Bassi N Padovani G Biondi N Bonini G Tredici MR Microalgae for oil: Strain selection, induction of lipid synthesis and outdoor mass cultivation in a low-cost photobioreactor Biotechnol Bioeng 2009 102 100 112 18683258 10.1002/bit.22033

[B48] Rao AR Dayananda C Sarada R Shamala TR Ravishankar GA Effect of salinity on growth of green alga *Botryococcus braunii* and its constituents Bioresour Technol 2007 98 560 564 16782327 10.1016/j.biortech.2006.02.007

[B49] Berges JA Franklin DJ Harrison PJ Evolution of an artificial seawater medium: improvements in enriched seawater, artificial water over the last two decades J Phycol 2001 37 1138 1145

[B50] Wan C Bai F-W Zhao X-Q Effects of nitrogen concentration and media replacement on cell growth and lipid production of oleaginous marine microalga *Nannochloropsis oceanica* DUT01 Biochem Eng J 2013 78 32 38

